# On the relation between a green and bright window view and length of hospital stay in affective disorders

**DOI:** 10.1192/j.eurpsy.2022.9

**Published:** 2022-02-22

**Authors:** Anna Mascherek, Sandra Weber, Kevin Riebandt, Carlos Cassanello, Gregor Leicht, Timothy Brick, Jürgen Gallinat, Simone Kühn

**Affiliations:** 1 Clinic and Policlinic for Psychiatry and Psychotherapy, University Clinic Hamburg-Eppendorf, 20246 Hamburg, Germany; 2 Lise Meitner Group for Environmental Neuroscience, Max Planck Institute for Human Development, 14195 Berlin, Germany; 3 Human Development and Family Studies, Pennsylvania State University, University Park, Pennsylvania 16802, USA

**Keywords:** Depression, greenness and brightness, length of stay, suppression effect, window view

## Abstract

**Background:**

The salutary effect of window views on greenery for inpatients in hospitals on length of stay and recovery rate has been repeatedly shown, however, not for psychiatric inpatients. The study assessed the association between a window view on green trees or man-made objects and brightness of the room on length of stay in a sample of psychiatric inpatients from one clinic.

**Methods:**

Data records of 244 psychiatric inpatients (mean age in years 41.8; SD = 11.8; 59.8% female, length of stay varying between 7 and 100 days) that were admitted between May 2013 and October 2018 with affective disorders were examined. Window view was assessed with images taken from each room and classified into showing man-made objects or green trees. The percentage of green within each image was also calculated as greenness of the view. Brightness was assessed with a luxmeter.

**Results:**

Although no effect was found for the dichotomous measures (man-made objects vs. green trees), a suppression effect emerged for percentage of green and brightness. The results indicate that both greenness of the window view as well as brightness significantly reduce length of stay in psychiatric inpatients with affective disorders.

**Conclusions:**

The suppression effect likely results from the characteristics of the windows; the greenest rooms also being the darkest. Due to the infrastructure of the ward, greenness and brightness came at the expense of each other. The results generally support the importance of a view into greenery and natural sunlight for recovery.

## Introduction

Literature on environmental psychology shows that exposure to natural environments exhibits salutary effects on human health and well-being [1–4]. The idea of environmental features systematically and significantly affecting human health and well-being is not new. In her work on standards of professional care, Florence Nightingale already pointed to the importance of daylight exposure, fresh air, and natural environments at the end of the 19th century [5]. And as of today, the importance of design and planning aspects in the context of patient care is well established. We also know that the salutary effect is not only limited to design or outside and outdoor experiences, but also presents itself in more subtle ways of exposure. The calming and salutary effect has been shown in studies that expose to both, real natural environments as well as to pictures displaying naturalistic sceneries [1–3]. The effect has been studied in healthy [1,3,6,7] as well as in clinical populations [8–10]. Kaplan and Kaplan’s Attention Restoration Theory [11] provides a theoretical framework for the effects, claiming that exposure to nature replenishes mental fatigue that is elicited by urban contexts. General requirements are that the environment must be fascinating, induce the feeling of being away, of extension and compatibility to exhibit beneficial effects.

In an influential study, Ulrich [12] related the perceived beneficial effect of natural environments to quantifiable differences in recovery rates of inpatients. He found shorter postoperative hospital stays and lower use of potent analgesics in a sample of surgical patients with windows in the hospital room facing a tree rather than a brick wall. In a quasi-experimental setting, he found scientific evidence that the mere aspect of differing window views significantly influenced recovery rates in an otherwise comparable sample. The effects reported refer to reduced stress and pain. Since then, this finding has been replicated in other somatic patient groups as well, for example, in women undergoing C-sections [13] or pulmonary and coronary patients. The effect was moderated by gender and diagnosis, with women reporting positive effects of a panoramic view on their subjective physical health and men rather reporting negative effects of a blocked view on mental health. The effect also differed between diagnostic groups, with the effect on mental health being more pronounced in pulmonary patients [14]. Generally, light and nature, even as exposure to artificial environments via virtual reality, have been shown to significantly reduce stress and pain in burn patients, patients undergoing a colonoscopy or heart surgery, and patients with myocardial infarction [15].

Depression is a highly prevalent mental illness that encompasses symptoms of stress and also pain in some patients. It has been observed that depressive patients benefit from exposure to the natural environment with respect to mood, anxiety, cognition, and attention [9,16]. Also, window views opening up to green space or natural environments are associated with lower risks of developing depression and anxiety [17], indicating that the beneficial effect does not necessarily depend on the physical interaction with nature. This leads to the idea of integrating window views as a simple, yet readily available resource accompanying state-of-the-art treatment of depressive patients to alleviate symptoms. Psychiatric inpatients usually experience longer and often even repeated stays as depression presents as a chronic condition in many individuals. The question of whether different window views exert a measurable effect on length of stay in depressive inpatients is interesting as it would represent a straightforward aspect that could easily be provided for every patient. Ultimately, even affecting length of stay would not only represent an interesting aspect for individual recovery but also from a more economical, public-health-perspective in terms of efficiency, even if the effect for the individual was small. However, to the best of our knowledge, Ulrich’s study has not been replicated in a sample of psychiatric patients, although other associations of design characteristics and psychiatric inpatients have been analyzed [18].

Another environmental aspect that has shown to be beneficial for depressive patients is light exposure to bright light (usually around 10000 lux), which is now an established additional procedure in the treatment of seasonal but also nonseasonal affective disorders [19,20]. Exposure to morning sunlight exhibits beneficial effects on bipolar inpatients with respect to length of stay [21]. Studies found that this effect is caused by bright artificial light as well as by sunlight [19,22,23], hence, suggesting that the effect is rooted in the amount of brightness rather than the exposure to actual sunlight. However, strong evidence for the utility of light therapy to *prevent* depression is lacking [24].

The aim of the present study was twofold. First, we wanted to replicate Ulrich’s [12] findings in a sample of depressive inpatients on a psychiatric ward. Hence, we investigated whether length of stay was impacted by the respective window view in a sample of psychiatric inpatients diagnosed with an affective disorder that was matched according to age range, window view, and time of year to the sample of patients reported by Ulrich [12]. We expected to find shorter periods of stay in patients who were located in rooms with a view onto green nature, most likely trees, instead of the “man-made” surroundings such as parking lots or buildings. Second, our analyses aimed at the notion that light/brightness exhibits positive effects on depressive patients in addition to the effect of greenness of the window view. We analyzed whether the assessed brightness of the room had an impact on length of stay, expecting greater brightness to be related to shorter stays, and also included a measure of greenness into this second analysis as we expected an additive effect of brightness and greenness.

## Methods

### Study design and participants

**Figure 1.**
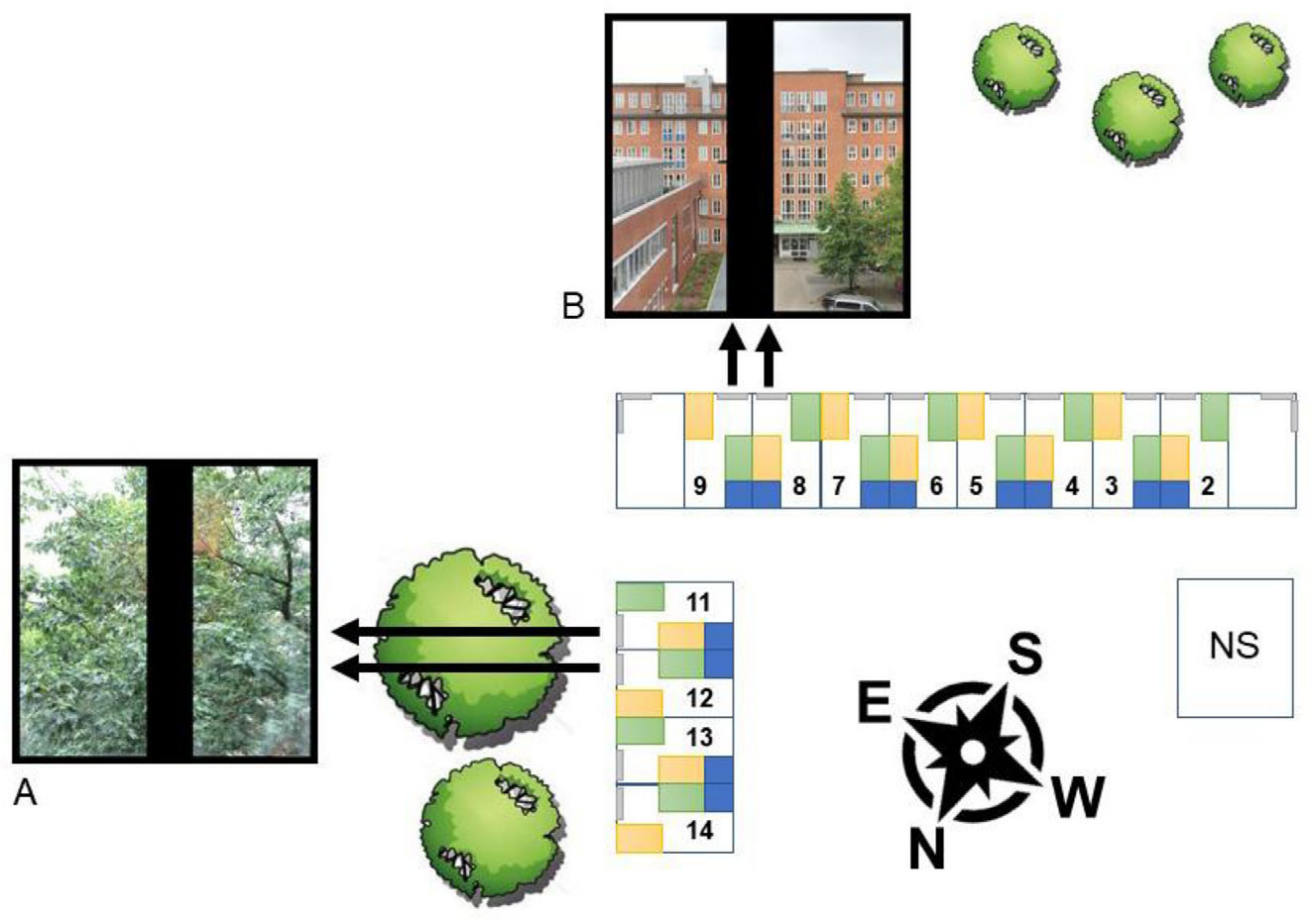
Location of the enclosed patient rooms and their distance from the nurses’ station (NS). (A) The window view from rooms 11/12. (B) The window view from rooms 8/9. Rooms 11–14 have an eastern orientation with a view of nature. Rooms 2–9 have a southern exposure with a man-made view.

Records of psychiatric inpatients treated for depression on a psychiatric ward of the University Medical Center Hamburg-Eppendorf, Germany, were obtained for the study. The sample consisted of records of patients admitted between May 2013 and October 2018, with length of stay varying between 7 and 100 days. All patients were located in rooms on the third floor of a four-story building of the hospital (see Figure 1). Twelve patient rooms were included in the study, with four of them having an eastern and eight of them having a southern orientation. Room 1 and 10 were excluded because these two rooms had two windows each. Patients who had a room change during their stay were not included. Treatment bias was prevented as the study was retrospective in nature and, hence, staff was not aware of the study. Every room had a window. All double rooms had their own bathroom and almost identical room size, design, and furnishing. Each window was 200 cm high and 200 cm wide with a middle bar of 23 cm. The height from the floor was 65 cm. All patients had an unobstructed window view which differed only by outdoor scenery. The patients were able to independently regulate the heating, tilt the window and draw the curtains. The artificial room lighting consisted of general room lighting and an integrated reading lamp above the bed. The patients could control both as they wished. There was no air conditioning. The rooms with the view on trees had an eastern orientation, the rooms with predominantly facing man-made objects had a southern orientation (see Figure 1). Patients were assigned to rooms mostly as they became vacant, with the exception of acute patients, who typically stay in the rooms close to the nurses’ station. To match the sample of the present study as precisely as possible with Ulrich [12], patients younger than 20 and older than 69 years of age were excluded. Also, only patients hospitalized between May and October were included. Overall, the sample comprised 244 inpatients. Mean age in years was 41.8 (SD = 11.8, age range 20–68 years), with 146 individuals (59.8%) being female. All patients were diagnosed with a depressive disorder according to the International Statistical Classification of Diseases and Related Health Problems (ICD-10). The severity of depression was coded according to ICD-10, ranging from a single episode, moderate depressive disorder (F32.1) to recurrent severe major depressive disorder with psychotic symptoms (F33.3). Frequencies of diagnoses are displayed in Table 1. Mean length of stay was 33 days (SD = 17.9 days, range 8–92 days). Patients staying less than 7 days were excluded, because it was assumed that they either suffered from an acute short crisis or were relocated to another ward. Finally, patients who stayed longer than 100 days were excluded, because their stay expands the usual maximal duration of roughly 12 weeks. The study was approved by the local psychological ethics committee of the University Medical Centre Hamburg-Eppendorf (LPEK-0075). Informed consent was not required.

**Table 1.** Frequencies of diagnoses according to ICD-10 and means and standard deviation for brightness and ratio of green pixel.

Variable			
	Number (%)	Mean ± standard deviation	
Diagnosis according to ICD-10			
Major depressive disorder, single episode, moderate (F32.1)	13 (5.3)		
Major depressive disorder, single episode, severe without psychotic features (F32.2)	72 (29.5)		
Major depressive disorder, single episode, severe with psychotic features (F32.3)	7 (2.9)		
Major depressive disorder, recurrent, moderate (F33.1)	4 (1.6)		
Major depressive disorder, recurrent, severe without psychotic features (F33.2)	133 (54.5)		
Major depressive disorder, recurrent, severe with psychotic features (F33.3)	15 (6.1)		
		Man-made	Tree
Green pixel density*		0.216 ± 0.08	0.401 ± 0.08
Brightness in lux		22117 ± 2757	1719 ± 376

*Note:* In our analyses, we chose major depressive disorder, recurrent, severe without psychotic features as our reference category as it represented the largest group.

* Numbers for ratio of green pixel represent relative frequencies.

### Measures

#### Man-made versus trees classification

To evaluate the content of trees and man-made features in each window view, pictures were rated by a convenience sample of 25 individuals on two dimensions. On one dimension, participants were asked to rate the content of trees on a continuous scale between “0” and “100.” The second dimension required a rating on the same scale with respect to man-made elements. Mean ratings across all participants were calculated. Ratings were then used to assign the window views to either the “tree” category or the “man-made” category. Eight window views were categorized as “man-made” and four as “trees.” Typical pictures of windows displaying views on man-made objects as well as of trees are shown in Figure 1. Window views classified as “tree” exhibited predominantly a view on treetops with foliage. The “man-made” views were characterized by views of parking lots and adjacent buildings. The trees in the tree images were located directly in front of the window, hence, blocking the full view of objects further away. The distance between the window sill and the first object outside was larger in the views characterized as “man-made,” allowing a view into the open. Although not formally measured, the difference in the openness of the view was persistent between the categories.

#### Brightness

We assessed brightness with a luxmeter from Dr. Meter (LX1330B). For this purpose, three measuring points were defined in the patient rooms, which had a distance of 1 m starting from the window going straight back into the room. We measured 1 m above the floor. Additional measurements were also taken to determine the brightest point in each room. This point varied in the different rooms. All measurements were carried out on September 16, 2020, between 11 and 11:30 a.m. We use the measurement of the brightest point in each room in our analyses.

#### Ratio of green pixels

In addition to the dichotomous categories of man-made objects versus trees, we assessed the ratio of green pixels in the photographs as a more quantifiable measure of green. Pictures of each window view were taken by a professional photographer in June 2019 between 10 and 11 a.m. with the automatic camera setting of an Iphone11 ProMax. The photos were cropped with an image processing program (Adobe Photoshop CS3, Version 10.0) to a pixel size of 2362 × 2362 px. Images were taken (and then adjusted as needed) so that the window in each photo spanned an identical number of pixels in the photo. Because the windows in all rooms were the same size, each pixel on any image represents the same amount of total window space. No color optimization was applied. The number of green pixels was then determined, as well as the green ratio for each photo. We applied Python programming language and the CV2 function library from the OpenCV package. All pixels of each image were examined individually. We then decided whether they were within the corresponding range of the HSV color space, which represents green (starting at *H* = 66°, *S* = 23.53%, *V* = 23.53% and ending at *H* = 160°, *S* = 100%, *V* = 100%). In CV2, this corresponds to the limits ranging from [33,60,60] to [80,255,255]. The percentage of green was estimated for each image (mean for green ratio: *m =* 26%, standard deviation = 12, ranging from 9 to 54% coverage). This percentage represents the total proportion of the image in terms of the color green, so it corresponds to the tree canopy. All remaining pixels not covered by this area represent colors that are not green according to the specified range of the HSV color space (see Supplementary Material for example images of our classification).

### Data analysis

We analyzed the data in a multiple linear regression framework using SPSS, version 26. We also analyzed our data within a time-to-event framework due to the structure of the data. Since the results were the same, we decided to report the results from the regression analyses as this is a more common notation in the field. A brief description of the time-to-event analyses is provided in the Supplementary Material. To assess the potential impact of window view over and above standard demographic variables, we entered sex, age, and diagnosis as predictor variables in a first step. Diagnoses were added as dummy-coded variables with recurrent depressive disorder, current episode severe without psychotic symptoms as reference. We chose this as a reference because it was the largest group in our sample and is commonly the most prevalent diagnosis for affective disorders on psychiatric wards. Data on smoking status and weight were taken into account in the study by Ulrich [12], however, were not available for the present sample. The variables man-made versus trees, brightness, and ratio of green pixels were then administered as variables of interest in two separate analyses. The first analysis only contained man-made versus trees to replicate Ulrich [12]. In the second analysis, brightness and ratio of green pixels were entered as the variables of interest.

## Results

Window view categories were correlated with the ratio of green pixels. A significant difference on mean level emerged for the window views (*t*_(242)_ = 15.63, *p* < 0.001, Cohen’s *d* = 2.16), with the tree-view exhibiting a larger proportion of green pixels as expected (see Table 1). Surprisingly, significant differences in brightness between rooms with a tree-view and rooms with a view onto man-made objects emerged (*t*_(197)_ = −96.7, *p* < 0.001, Cohen’s *d =* 8.6; Table 1), with man-made objects exhibiting higher brightness.

### Length of stay and window views

First, we analyzed whether length of stay differed as a function of window view. Parameter estimates are displayed in Table 2. Results show that differences in diagnoses were only significant for recurrent depressive disorders as well as the current episode severe with psychotic symptoms. Although this could be predicted from the nature of the disorder, one has to consider that lack of significance might also reflect a lack of power due to the small size of the subgroups. Age and sex did not exhibit a significant effect. There were no significant interaction terms between sex and diagnosis. For our main variables of interest, we found an unexpected trend (on *p* = 0.09) toward a shorter stay of patients with window view toward man-made objects. Cohen’s *f* ^2^ of 0.012 pointed to a small effect.

**Table 2.** Standardized parameter estimates for length of stay in linear regression models.

Variable	Unstandardized coefficients (standard error)		
	Tree versus man-made	Brightness	
		Model I	Model II
Age in years	0.024 (0.097)	−0.002 (0.098)	0.009 (0.97)
Sex^a^	0.83 (2.3)	0.90 (2.3)	0.831 (2.3)
Diagnosis^b^			
F32.1	−8.4 (5.1)	−8.86 (5.2)	−7.68 (5.1)
F32.2	−0.05 (2.6)	0.012 (2.6)	−0.17 (2.6)
F32.3	−7.6 (6.9)	−8.15 (6.9)	−6.43 (6.9)
F33.1	−10.6 (9.0)	−11.91 (9.0)	−10.15 (9.0)
F33.3	−10.2* (4.8)	10.5* (4.8)	10.1* (4.8)
Environmental variables			
Tree versus man-made^c^	−4.5 (2.6)		
Ratio of green pixel		−0.001 (0.002)	−0.008* (0.004)
Brightness			−0.460* (0.2)

Abbreviation: *CI*, confidence interval.

* *p* < 0.05.

a With category “female” as reference.

b Diagnoses are entered as dummy-coded variables. We chose major depressive disorder, recurrent, severe without psychotic features (F33.2) as our reference category as it represented the largest group.

c With category “tree” as reference.

### Length of stay and brightness

**Figure 2.**
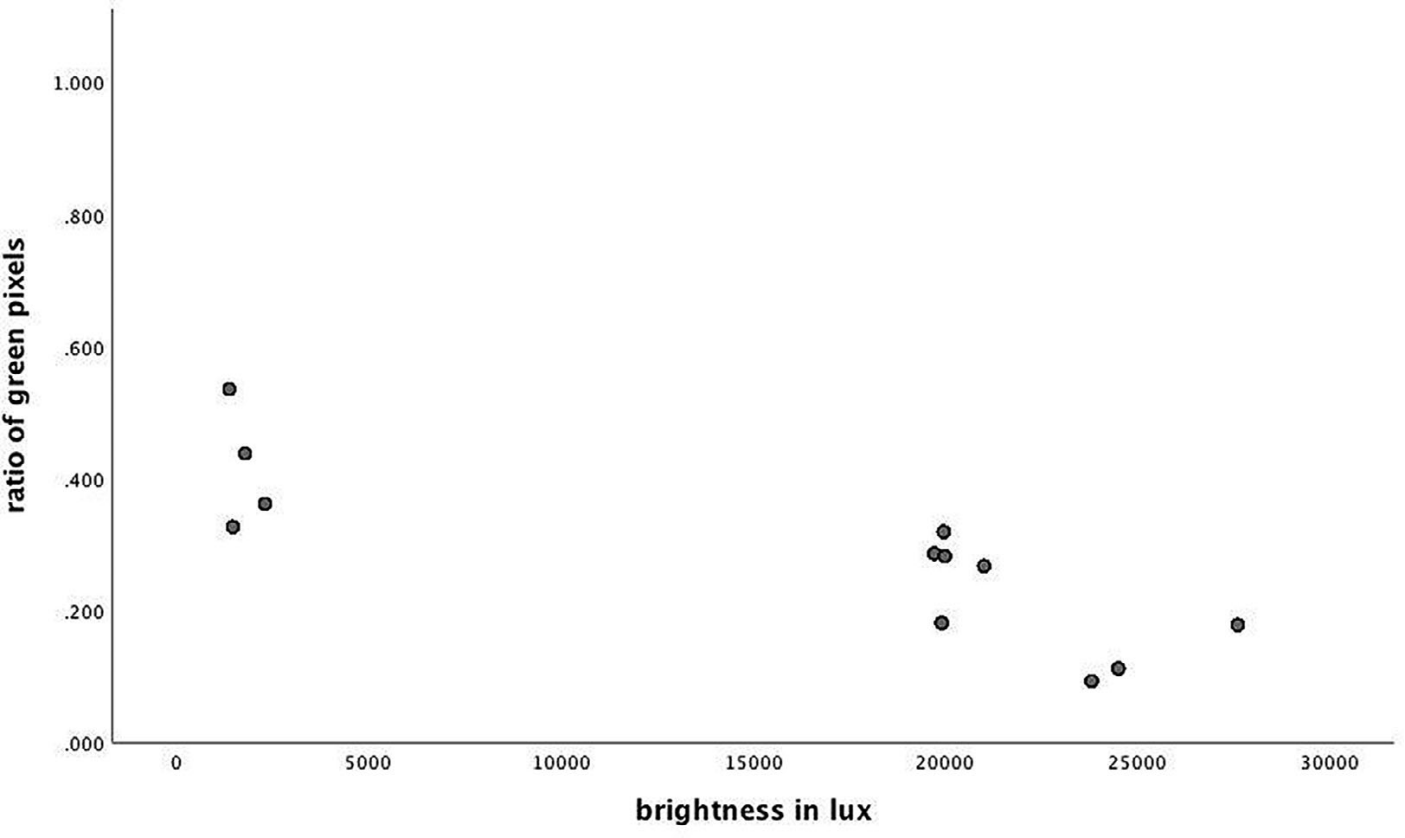
Scatterplot of the distribution of brightness and greenness across window views. Brightness is measured in lux, ratio of green pixels is displayed in relative frequencies.

In our second analysis, we analyzed the potential effect of brightness on length of stay. The same covariates as in our first analysis were added. We replaced the categorical classification of the window views with a continuous covariate given by the ratio of green pixel in the image taken from the window view to account for the categories man-made versus trees. Greenness did not exhibit any significant effect (*β* = −0.019, *p* = 0.77). However, upon adding brightness as a variable of interest in this analysis, we found an unexpected significant effect for both brightness and ratio of green pixels, which indicates a suppression effect. The suppression effect suggests that both, greenness and brightness contribute significantly. The suppression effect likely results from the characteristics of the windows in our study. Specifically, the greenest rooms were often the darkest because the windows sit in the shadow of the trees; by contrast, the brightest rooms (where fewest trees obstruct the light) had the least green. This tradeoff can be seen in the examples in Figure 2. The effects of high greenness are reduced by the effects of low brightness and vice versa, although when both are included, the model contains enough variability to separate their individual effects. There was no significant interaction between greenness and brightness. Both parameters (brightness: *ß* = −0.24, *p* = 0.020 and ratio of green pixels *ß* = −0.20, *p* = 0.046) exhibited a significant negative effect on length of stay, indicating that brightness as well as greenness reduced the length of stay in psychiatric inpatients with affective disorders. The results in our sample for the effect of greenness read as follows: one standard deviation increase in greenness corresponded to −0.2 standard deviation decrease in length of stay, holding brightness constant; similarly, one standard deviation increase in brightness corresponded to −0.24 standard deviation decrease in length of stay, holding greenness constant.

## Discussion

In this article, we first aimed at replicating the relation between length of stay and window view (man-made vs. trees) from Ulrich’s [12] seminal work in a sample of psychiatric inpatients. Psychiatric samples represent a group of individuals often experiencing longer and repeated stays, making it especially interesting to learn about their potential reactions to respective window views. Second, we analyzed the potential effect of room brightness on length of stay as exposure to light has been found to exhibit salutary effects in patients with affective disorders. We will discuss our findings in what follows.

### Window view

We found no significant effect for the different window views consisting mostly of trees or of man-made objects, that is, parking lot and adjacent buildings. Hence, we could not replicate the findings of Ulrich [12]. Three possibilities present themselves as reasons for this lack of replication. One explanation might simply be a limitation of power, although this seems unlikely given that our sample was almost 10 times the size of the sample in Ulrich’s [12] study. Second, it is possible that our experimental setup was sufficiently different: it is possible that psychiatric patients are less receptive to the effects of their surroundings, or that because our sample was not bedridden and actual time spent in the room or even in bed could not be controlled for in our analyses, participants might simply have had too little exposure to the view. However, both of these possibilities are somewhat unsatisfying, although future work should still address them. In particular, these are undermined by the nonsignificant trend toward shorter periods of stay in patients looking out on man-made objects. This trend directly contradicts Ulrich’s finding at first glance.

We propose a more nuanced view. Indeed, a recent review by Barnes et al. [25] identified a variety of features contributing to the effects of green spaces, indicating that green does not equal green at all times. They reviewed the “nature of the nature” in 30 studies reporting beneficial effects on mental health and well-being. They generally criticize the level of detail as not satisfactory, point out, however, that “beneficial nature” contained greenery, trails, and water. The authors conclude that in order to really extract the features of natural environment that are beneficial, much more attention has to be paid to (the description of) details. Man-made objects, for example, are not per se detrimental; and size of a park is not per se beneficial; it rather seems to depend on the mix of specific environmental features. In the study by Ulrich [12], windows in the man-made condition faced a rather solid brick wall, while those in the nature condition viewed open greenery. This suggests that Ulrich’s conditions confounded several constructs; natural views contained nature, but also brightness, greenness, and expanded space perception, while man-made views contained none of those. It has been repeatedly found that both the exposure to light as well as to natural environment exerts beneficial effects on patients with affective disorder [21,22,26,27]; expanded space perception has also been found to exert its own effect on health and impulsivity [28].

Looking closely at the specific pictures and features of the view in our sample, we found that the view toward man-made structures was often accompanied by *greater* brightness, albeit low levels of greenness. By contrast, all trees were rather close to the window and covered with lush summer foliage. As a result, views containing the natural scene of a tree in front of the building were highly green but also turned out to be the darkest rooms because the vegetation prevented the daylight from illuminating the room. Our results suggest that rooms with windows facing trees had reduced brightness and vice versa, leading to a less clear differentiation of more and less salutary influences. To disentangle these effects and to potentially replicate the finding from Ulrich’s study, window views that are both green and bright, as well as window views that contain man-made features and are dark would be needed. Our second analysis was able to shed some extra light on the results of the window view effects.

In our second step, we analyzed the potential effect of brightness in the room on length of stay. Using the ratio of greenness as the only predictor, no significant effect emerged. However, when brightness was added as an extra predictor, both variables exhibited a significant effect, with both brightness and green being associated with shorter periods of stay. Statistically, our results describe a suppression effect. Here, the suppression is a side-effect of the negative correlation between green and brightness in our sample. The brightest rooms were also the rooms with the least exposure to green, and so the positive effect of increased brightness in a room was reduced by the negative effect of lower greenness. Both variables appear to have true effects on length of stay; the suppression effect makes it difficult to statistically discern the effects of either one without controlling for the effect of the other.

Our data suggest that both brightness and greenness exhibit a positive effect on the recovery of inpatients with affective disorders. It has been shown that the positive effect of brightness on depression is true for sunlight as well as artificial light [19,21,22,29]. It remains an interesting open question, whether the combination of artificial bright light in rooms with a view on trees would have exerted the most beneficial effect on length of stay in our sample. The question remains whether the darkness from the foliage could be compensated by artificial light. This would point toward a true effect of physical light and not an effect of *perceived* darkness/brightness of a room or the limitations of space perception evoked by the foliage. Also, it remains an open, yet interesting question, whether the effect of artificial greenness might be able to compensate man-made objects in individuals with affective disorders. We assessed brightness at around autumnal equinox as a proxy for brightness covering the whole time span of our study. A further question remaining is whether the effects found in the present study might even be more pronounced around solstice, that is, when days are longer. Future studies should take this into consideration. Another aspect that would be interesting to assess is the inclusion of the unobstructed view of the sky and thus the consideration of the proportion of blue pixels in the images.

Another interesting point that goes widely unnoticed is whether or not the characteristics of the window view also exert their influence by night. We are not aware of any study systematically assessing the potential effect of window view by night. However, studies exist that point to a general association between depression and artificial night light (e.g., [30,31]), hence, we consider that an interesting aspect. This might be especially interesting in a sample of psychiatric inpatients, who might be often suffering from insomnia. It is plausible to assume that other characteristics such as lighting, noise, or temperature might be more important at night than the actual view. This opens up an area of questions worthwhile to be addressed.

Concerning the size of the effect in our study, an increase of 1000 lux was associated with a decrease of roughly half a day’s stay, although presenting a rather broad confidence interval with estimates ranging between an unstandardized coefficient of *b* = −0.85 at the lower bound and *b* = −0.07 at the upper bound. As admission and discharge were not assessed in terms of the time of day, but only as date, some additional imprecision is inherent to our dependent variable. The same precaution applies to interpreting the effect of green pixels. Translated into a reduction of time spent on the ward, our analysis shows that an increase of 1000 green pixels was associated with a decrease of 0.008 days. Although we caution the reader against using these numbers as precise estimates in practical settings, these analyses suggest that both natural environment exposure and sufficient illumination of patient rooms must be considered when conceptualizing patient wards as they exert salutary effects on inpatients and might ultimately even be translated into economic benefit. The small numbers for each patient might mask the implications the results could exert if integrated systematically and comprehensively in patient care. As depressive inpatients cause millions of days spent in hospitals, even a reduction of a fraction of a day in every patient would sum up to a substantial amount of money and time saved on societal level. However, we are eager to stress that our results may serve as a hint toward the relevance of window view in psychiatric inpatients, but may not be taken as a calculation basis for actually estimating costs or time saved.

The current study has some limitations. In our study setting, exposure to green environments came at the expense of brightness and vice versa. Future studies should ideally analyze these effects in a design where the predictors are not inversely related to assess potential additive effects as well. Variables and important covariates that are known to influence treatment success and therefore length of stay, such as status of repeated hospitalization or not, smoking habits, general health status, comorbidities, treatment, or socioeconomic status were neither available nor included. This is due to the fact that our data were made available from the hospital administration, where only data encoding the general treatment conditions are assessed. Future studies should ensure a more detailed characterization of the sample to further assess the relevance of the present results. This is true for the additional basic sociodemographic variables referred to above, but also for self-reported data on, for example, mood, stress, or other symptoms relevant in affective disorders. Furthermore, this study took place on a psychiatric ward where patients were supposed to be in bed only during the night. Any potentially confounding effect of actual time of exposure to both brightness of the room and view remains uncontrolled in the present study. This is in contrast to Ulrich’s study [12], since he assessed surgical patients after cholecystectomy, who presumably required bed rest. The limited number of rooms poses another constraint, although this shortcoming is not unique to our study, since rooms in wards and hospitals are naturally restricted. Also, the suppression effect found in the present study deserves further studying in order to understand its mechanisms and driving forces. It remains an open question, whether the distance between the window sill and tree/man-made object had a confounding effect. This could not be entangled with the data of the present study, as distances were systematically shorter in the tree window views and larger in the man-made object window views. However, this aspect should be considered in future studies.

To summarize, our results suggest that for patients with affective disorders, not only the window view of a natural scenery exerts a salutary effect but also accommodation in a bright room is important. Further research is needed to further examine this finding.

## Data Availability

The data are available from S.K. upon request.

## Supplementary Materials

To view supplementary material for this article, please visit http://doi.org/10.1192/j.eurpsy.2022.9. click here to view supplementary material
