# Supplementary material for: On the relation between a green and bright window view and length of hospital stay in affective disorders
Source: Eur Psychiatry. 2022 Feb 22;65(1):e21. doi: 10.1192/j.eurpsy.2022.9 (PMC8988271; doi:10.1192/j.eurpsy.2022.9)
Supplement: Supplementary file 1 [file S0924933822000098sup001.docx]

**On the relation between a green and bright window view and length of hospital stay in affective disorders**

Anna Mascherek^1^, Sandra Weber^1^, Kevin Riebandt^1^, Carlos Cassanello^1,2^, Gregor Leicht^1^, Tim Brick^3^, Jürgen Gallinat^1^, Simone Kühn^1,2^

^1^ University Clinic Hamburg-Eppendorf,

Clinic and Policlinic for Psychiatry and Psychotherapy,

Martinistraße 52, 20246 Hamburg, Germany

^2^ Max Planck Institute for Human Development,

Lise Meitner Group for Environmental Neuroscience,

Lentzeallee 94, 14195 Berlin, Germany

^3^ Penn State,

College of Health and Human Development,

231 Health and Human Development Building

University Park, PA 16802, USA

### Corresponding author: Simone Kühn

Max Planck Institute for Human Development

Lentzeallee 94, 14195 Berlin, Germany

e-mail: [kuehn@mpib-berlin.mpg.de](mailto:skuehn@cbs.mpg.de)

**keywords**: depression, length of stay, window view, greenness and brightness, suppression effect

*Time-to-event-analyses*

The structure of the data also warrants the analyses in a time-to-event-framework. Discharge can be classified as “event”, hence, the time until discharge can be modelled.

In our analyses, time-to-event is defined as time until discharge. We only included patients with length of stay between 7 and 100 days. Patients with lengths of stay longer or shorter were excluded from the analyses (see main manuscript for explanation).

We included the same independent variables and covariates as in the standard regression model.

In a first step, we calculated Kaplan-Meier plots for patients with windows facing trees and patients facing a view with man-made objects. Results are shown in figure 1.


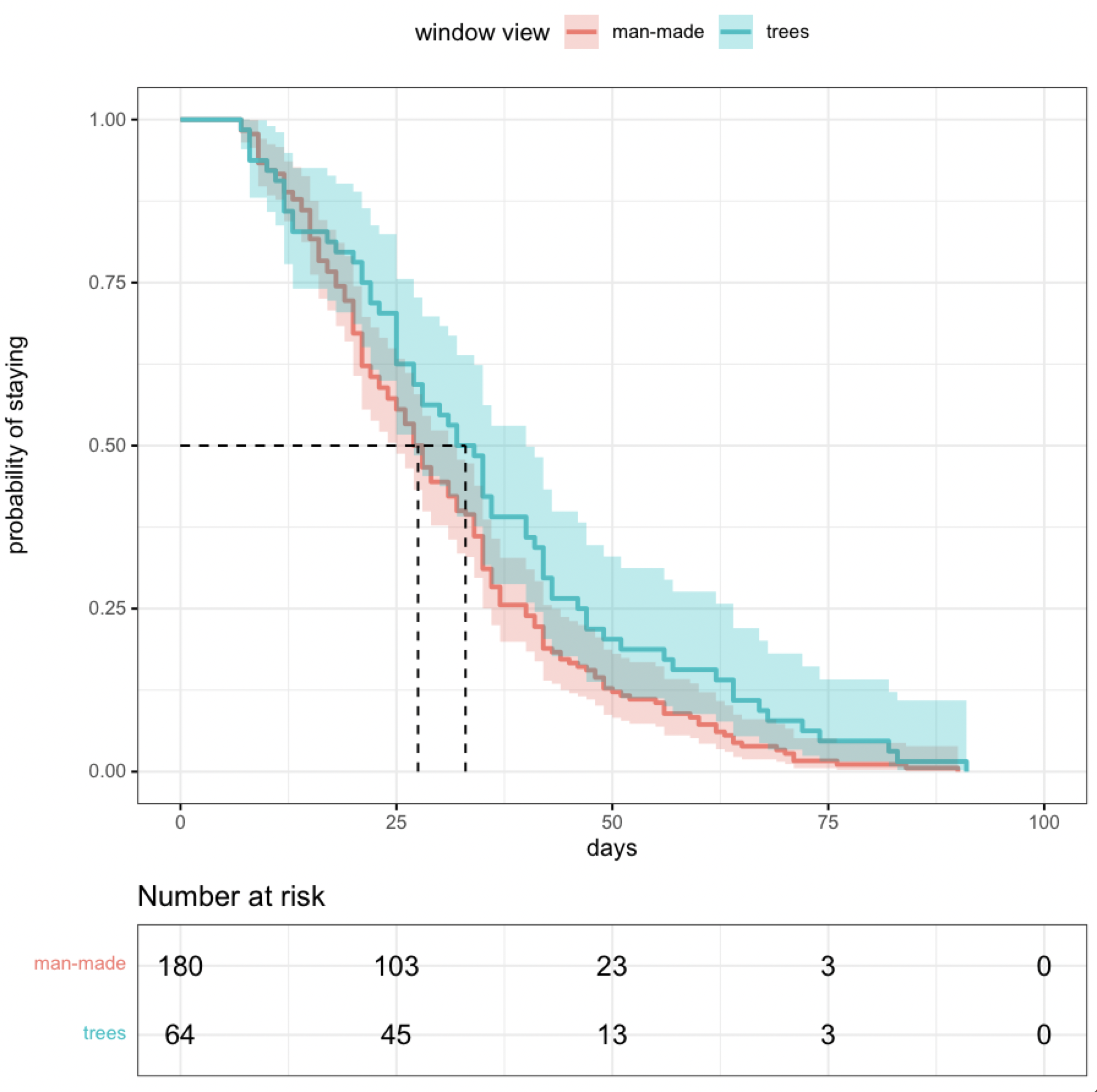


Figure 1. Kaplan-Meier plots of probability of staying in patients with a window-view facing trees and patients with a window facing man-made objects.

In a second step we ran a cox proportional hazards regression analysis with the results shown below in table 1. Our independent variables of interest were the ratio of green pixel and brightness of the room. We also included the same covariates as in the standard regression analysis, namely, age, sex, and diagnosis.

Table 1. Cox Proportional Hazards Estimates of the Determinants of length of stay in a sample of psychiatric inpatients (*N* = 244).

| Variable | Coeff | se(coeff) | z-statistic |
| --- | --- | --- | --- |
| Sex (female)^a^ | -.06 | .14 | -.42 |
| Age | .0002 | .005 | .03 |
| Diagnosis^a, b^ (F33.2) |  |  |  |
| F32.1 | .62 | .30 | 2.09** |
| F32.2 | -.007 | .15 | -.05 |
| F32.3 | .51 | .39 | 1.297 |
| F33.1 | .99 | .52 | 1.9* |
| F33.3 | -.49 | .28 | -1.78* |
| Brightness | .00003 | .000012 | 2.541** |
| Green Pixel | .22 | .11 | 2.042** |

Note: ^a^ Reference category in parenthesis; ^b^ affective disorders were diagnosed according to the International Statistical Classification of Diseases and Related Health Problems (ICD-10). Severity of depression was coded according to ICD-10, ranging from a single episode, moderate depressive disorder (F32.1) to recurrent severe major depressive disorder with psychotic symptoms (F33.3); ** = p < .05, * < .1


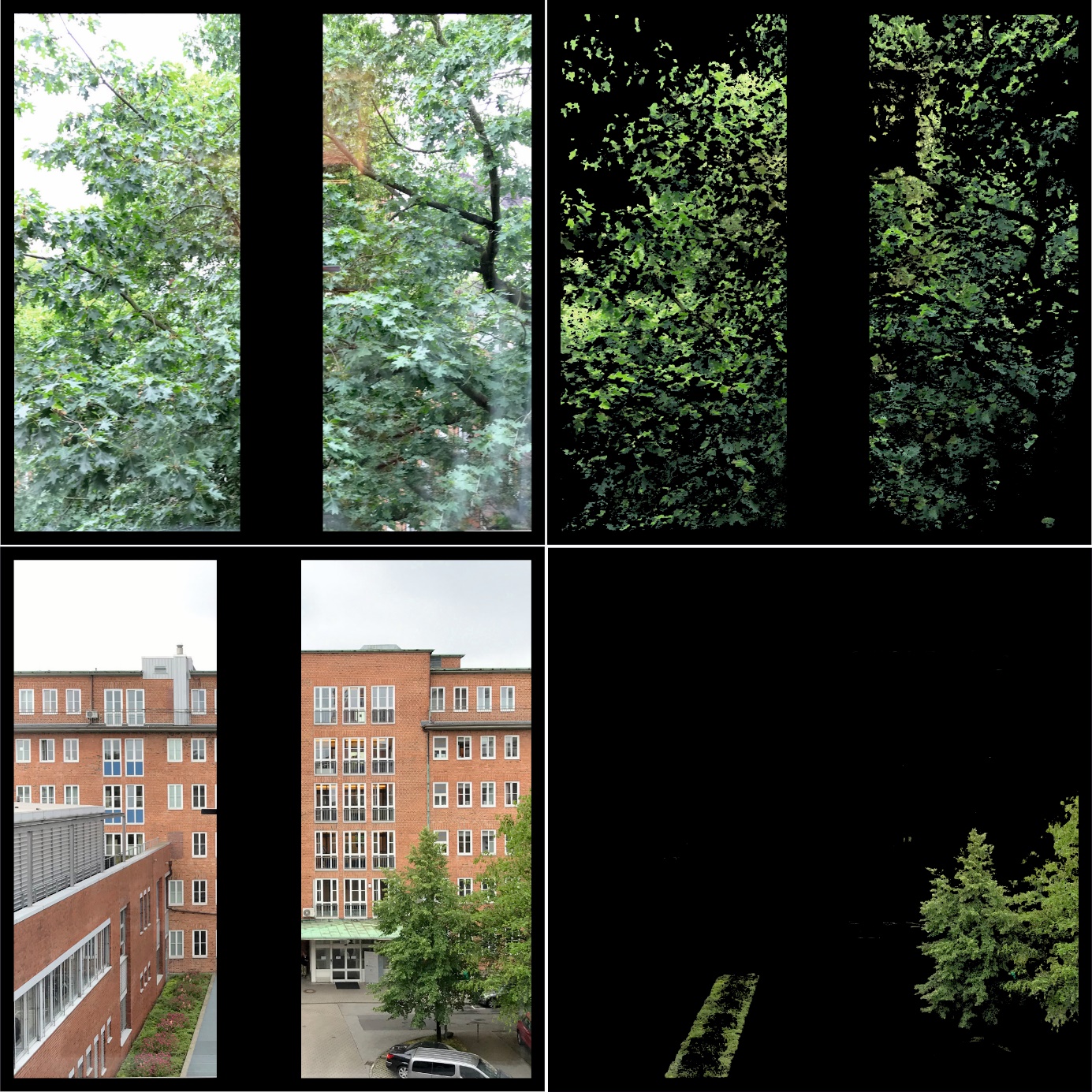


Figure 2. Examples of how the algorithm classified green in the image. On the left, the original photo is shown in each case, while on the right, only the area classified as green is shown.
